# Introduced Siberian Chipmunks (*Tamias sibiricus barberi*) Contribute More to Lyme Borreliosis Risk than Native Reservoir Rodents

**DOI:** 10.1371/journal.pone.0055377

**Published:** 2013-01-31

**Authors:** Maud Marsot, Jean-Louis Chapuis, Patrick Gasqui, Anne Dozières, Sébastien Masséglia, Benoit Pisanu, Elisabeth Ferquel, Gwenaël Vourc’h

**Affiliations:** 1 INRA, UR346 Epidémiologie animale, Saint Genès Champanelle, France; 2 Muséum National d’Histoire Naturelle, Département Ecologie et Gestion de la Biodiversité, UMR 7204 Conservation des Espèces, Restauration et Suivi des Populations, MNHN-CNRS-P6, Paris, France; 3 Institut Pasteur, Equipe des Borrelia, Paris, France; University of Minnesota, United States of America

## Abstract

The variation of the composition in species of host communities can modify the risk of disease transmission. In particular, the introduction of a new host species can increase health threats by adding a new reservoir and/or by amplifying the circulation of either exotic or native pathogens. Lyme borreliosis is a multi-host vector-borne disease caused by bacteria belonging to the *Borrelia burgdorferi* sensu lato complex. It is transmitted by the bite of hard ticks, especially *Ixodes ricinus* in Europe. Previous studies showed that the Siberian chipmunk, *Tamias sibiricus barberi*, an introduced ground squirrel in the Forest of Sénart (near Paris, France) was highly infested by *I. ricinus*, and consequently infected by *B. burgdorferi* sl. An index of the contribution of chipmunks to the density of infected questing nymphs on the vegetation (i.e., the acarological risk for humans) was compared to that of bank voles (*Myodes glareolus*) and of wood mice (*Apodemus sylvaticus*), two known native and sympatric competent reservoir hosts. Chipmunks produced nearly 8.5 times more infected questing nymphs than voles and mice. Furthermore, they contribute to a higher diversity of *B. burgdorferi* sl genospecies (*B. afzelii, B. burgdorferi* sensu stricto and *B. garinii*). The contribution of chipmunks varied between years and seasons, according to tick availability. As *T. s. barberi* must be a competent reservoir, it should amplify *B. burgdorferi* sl infection, hence increasing the risk of Lyme borreliosis in humans.

## Introduction

Changes in host communities are suspected to be one of the key parameters influencing the emergence of diseases caused by multi-host pathogens [1]. Indeed, changes in species abundance and community composition modify the ability of host populations to transmit pathogens, e.g. the encounter rate between susceptible and infected individuals, and/or the probability of transmission when an encounter does take place [2]. Thus, to improve our understanding of how the risk of multi-host diseases is associated with host community changes, the role of the different host species in the transmission dynamics of pathogens needs to be characterized [3]. This is particularly complex for vector-borne diseases because three actors - the pathogens, the vectors and the hosts – are involved in the transmission dynamics. Three main methods have been developed to quantify the contribution of host populations to the circulation of multi-host pathogens of tick-borne diseases. First, host contribution has been estimated through the evaluation of the basic reproduction number, R0 [4], [5]. R0 is defined as the average number of secondary cases arising from one infectious individual in a population consisting entirely of susceptible individuals. Many important parameters of these models are considered constant (e.g. host density), and many are defined from the literature. Two examples are the coefficient of transmission between ticks and vertebrates, and the coefficient of tick survival from larval to nymph stage, both of which are crucial in the transmission dynamics [5]. The models would be more robust if the parameters took into account key variations (e.g. host dynamics), and could work on estimates from field data. The second method used to quantify host contribution was developed by Brisson et al [6], who estimated the part of the risk originating from a given species by assigning pathogens found in the source of the risk (*Borrelia burgdorferi* in questing ticks) to a given host species. The third method, proposed by Mather et al [7], developed an index of contribution for each host species, known as the “reservoir potential”, based on field data. Remarkably, none of these studies took into account the sources of variation of the contribution by each host species.

The introduction of host species is one of the major causes of changes in host communities [8], and thus of the emergence of diseases [9]. Several studies have reported the introduction of new pathogens due to the introduction of their hosts [10], [11]. Surprisingly, the possibility of an introduced host species amplifying a local pathogen has received little attention [12]. This is probably because the influence of an introduced species on local pathogen dynamics is difficult to evaluate when the dynamics before introduction are not known. To our knowledge, the only corresponding example is the increase of Buggy Creek virus prevalence in cliff swallows (*Petrochelidon pyrrhonota*) following the introduction of the house sparrow (*Passer domesticus*) in Nebraska [13].

In this paper, our objective was to evaluate the contribution of an introduced species, the Siberian chipmunk (*Tamias sibiricus barberi* Johnson and Jones 1955 [14]), to the risk of the most prevalent vector-borne disease in Europe, Lyme borreliosis [15]. The introduced Siberian chipmunk is a Sciurid native to Korea [16] which has been sold in European pet shops since the 1960s, and intentionally released into the wild since the 1970s [17]. Since then, 22 populations in Europe, and 11 in France, have been identified in forests and urban parks [18].

Lyme borreliosis is caused by pathogenic bacteria belonging to the *Borrelia burgdorferi* sensu lato (sl) complex [19]. These bacteria are transmitted by the bite of hard ticks, especially the *Ixodes ricinus* species in Europe. The main genospecies present in Europe that are pathogen for human are *B. afzelii*, that infect preferentially rodents, *B. burgdorferi* sensu stricto (ss), found in birds and mammals, and *B. garinii* that infects preferentially birds [20]–[22]. The risk for humans to be infected by Lyme borreliosis agents is measured by the density of infected questing nymphs on the vegetation, called the acarological risk. Siberian chipmunks are suspected of contributing greatly to Lyme borreliosis risk because they host more ticks and are more infected by more diverse genospecies than local rodent reservoir species [23], [24]. We evaluated the contribution of a host population to Lyme borreliosis risk by calculating the production of infected questing nymphs by this host based on the “reservoir potential” proposed by Mather el al [7], and by taking into account different sources of variation of host contribution to the acarological risk. We did so for *B. burgdorferi* sl and for the different genospecies. First, we compared the contribution of the Siberian chipmunk to that of two native reservoir rodent species, the bank vole (*Myodes glareolus*) and the wood mouse (*Apodemus sylvaticus*). Second, we investigated the variation of the contribution of the Siberian chipmunk according to time and seasons. These are factors known to influence host tick burden and infection prevalence in *B. burgdorferi* sl [25], which are both components of host contribution.

## Materials and Methods

### Study Site

The study was conducted in the forest of Sénart (3,200 ha, more than 3 million visitors per year [26]), located 22 km southeast of Paris. Siberian chipmunks were first introduced in the northwest section of the forest in the 1970’s [17], and began to settle on the study site 5 km further to the south-east in the early 2000’s [27]. The forest now holds the largest population of chipmunks known in France [18]. In this forest, the rodent community consists mainly of Siberian chipmunks, of bank voles and of wood mice. Red squirrels (*Sciurus vulgaris*) and field voles (*Microtus agrestis*) are scarce on the study site.

### Host Capture

All conducted experiments complied with the current laws of France. Introduced Siberian chipmunks were trapped monthly on a 14 ha area (La Faisanderie: 48°39′25″N, 2°29′40″E) between March and October over a four-year period (2007 to 2010). A grid made by 104 geo-localized Sherman© traps baited with peanut butter and sunflower seeds was set from sunrise to sunset [27]. In 2007, two trapping sessions, one lasting three consecutive days, the second five consecutive days, were performed during each study month at 15-days intervals [27]. A five-day long trapping session then was conducted every month from 2008 to 2010 [24]. One ear biopsy, used to detect *B. burgdorferi* sl in blood and tissue, was obtained from each chipmunk [28] by cutting a small piece (maximum of 3 mm^2^) from the ear with scissors. The tissue was stored immediately in 90% ethanol. Only chipmunks captured for the first time during the course of the study were analysed. During the examination of the chipmunks, we counted the tick larvae found on the head [29], [30] using eye lenses (3×magnification).

Bank voles were caught regularly on the trapping grid (see [24]). Up to 30 voles were euthanized monthly by cervical dislocation between 2007 and 2008, immediately put into a plastic bag, and frozen for later analysis in the laboratory. Wood mice were sampled monthly only in 2007, using Sherman traps placed outside the trapping grid set on La Faisanderie, but in a similar habitat and in a sympatric population, initially devoted to the study of the natal dispersion of chipmunks [31]. An ear biopsy for voles and mice was obtained following the same procedure described for chipmunks to detect the presence of the bacteria in blood and tissue.

### Tick Counts

Counts of larvae on chipmunks were realized in the field, and only done on the head [29]. Larvae on bank voles and wood mice were collected from the entire body and, counted and identified to species level under an optical microscope in the laboratory. *I. ricinus* represented more than 90% of identified specimens. To have comparable data between small rodents and chipmunks, we developed a model to estimate the total number of larvae on a chipmunk’s entire body, using larvae counts realized at the same time in the field (head count) and in the laboratory (body count). This was realized on 19 euthanized chipmunks that were obtained from a study in another French periurban forest, Verneuil-sur-Seine (Yvelines), in 2007. A regression model was done between the larvae number counted in the field and the larvae number counted in the laboratory [32]. From the hypothesis that there is a linear relation between larvae counts in the field and in the laboratory, the initial count variables of field and laboratory (respectively Y_F_ and Y_L_, which follow a Poisson distribution), are represented by the square root of these counts (respectively Z_F_ and Z_L_), which follow a normal distribution [33]. We obtained the following relation: Z_F_^i^ = √ α.Z_L_^i^+e^i^, with α the correction coefficient of larvae counts on the field equal to 0.7 (Appendix S1).

### *Borrelia* Molecular Identification

DNA from one ear biopsy per chipmunk, vole and mouse were extracted using NucleoSpin® Tissue kit (Machery-Nagel, Düren, Germany). The presence of *B. burgdorferi* sl in the extracted DNA was detected using a PCR that targets the 16S rRNA gene with [5′-ATGCACACTTGGTGTTAACTA-3′ (819–842] and [3′-GACTTATCACCGGCAGTCTTA-5′ (1153–1173)] primers [34]. *B. burgdorferi* sl species were identified on positive PCR products using a PCR that targets the intergenic *rrf-rrl* spacer followed by *Mse*I restriction pattern of products amplified with primer 1 (5′-CTGCGAGITCGCGGGAGA-3′) and primer 2 (5′-TCCTAGGCATTCACCATA-3′) [35]. This method does not differentiate *B. garinii* from the newly identified species in rodents *B. bavariensis* [36].

### Density of Rodents

Siberian chipmunk densities were calculated for three months (March, June and September) between 2007 and 2010, corresponding to spring, summer and autumn. These periods reflected chipmunk population biology, with the main emergence of adults from their hibernation burrow occurring in March, the emergence of J1 from the birth burrow in May-June, and that of J2 in September [37]. Densities of chipmunks were estimated by fitting spatially explicit models to the animal trapping data implemented in the package ‘secr’ in R [38], [39]. Home-range centers were assumed to be Poisson distributed, and detection function followed a half-normal curve, where capture probability decreases with the distance to the trap. As no trap saturation was observed during the entire study period, a maximum likelihood (ML) method [40], [41] was used. The spatial boundary strip was set at 100 m after checking that estimates of density did not vary with increased width. The spacing for the integration mesh of the ML estimator was set to 32 × 32 points, matching the contour of the trapping grid. The optimization algorithm was set to the ‘BFGS’ method because the default ‘Newton-Raphson’ algorithm most often failed to compute the information matrix. The default values implemented in the package otherwise were used for all of the computations. We ran models using all of the data according to trapping sessions (n = 32). We ran models with constant parameters, and tested for an influence of site-specific learned response that we noted as “bk” [38], corresponding to trap-happy or trap-shy behavior at a given trap, affecting each parameter of the model, i.e., detection probability noted g(0) and movement scale noted σ. The best model was selected by considering the Akaike’s information criterion, corrected for small sample size. A conditional likelihood incorporating the different trapping sessions was used to derive densities.

For bank voles and wood mice, densities were estimated from marked-recaptured individuals caught over three consecutive days in 100 baited INRA© live-traps spaced approximately 3 m apart, distributed along 2 lines and added to the trapping grid in March, June and September [42], [43]. Densities for voles and mice could not be estimated by ‘secr’, because we did not geo-localize the INRA traps, nor note the trap where an individual was caught. We estimated population abundance using the Robust Removal model M*_bh_* of Pollock and Otto [44] implemented in the CAPTURE program [45]. The abundance obtained was divided by an effective trapping area of about 3 ha, corresponding to a boundary strip 25 m wide and 600 m long lying along the trapping lines. The boundary strip width was chosen according to published estimations of home range size and rodent movements [46]–[48].

### Statistical Estimation of Larvae Burden and Infection Prevalence

To evaluate the contribution of chipmunks between 2007 and 2010, voles in 2007 and 2008, and mice in 2007, for three seasons in the year (spring = March – April, summer = May – June – July, and autumn = August – September – October), we used estimates of larvae burden and *B. burgdorferi* sl infection prevalence. Mean larvae burden was estimated per host species with Generalized Linear Models (GLM) [49] using a negative binomial distribution (log link) with season and year as explanatory variables. We also estimated seasonal and yearly infection prevalence by *B. burgdorferi* sl and by each *Borrelia* genospecies, per host species from data of ear biopsies with a GLM using a binomial distribution (cloglog link) with season and year as explanatory variables. Using the standard errors of each corresponding model, 95% confidence intervals were calculated for the estimations of mean larvae burden and mean infection prevalence. All analysis programs were written with R software (R Development Core Team, 2008).

### Index of Contribution

The index of contribution to Lyme borreliosis risk that we estimated depended on host density and larvae burden and the prevalence and infectivity of *B. burgdorferi* sl and of each genospecies. To estimate an index of contribution, we modified the “reservoir potential” developed by Mather et al. [7]. The “reservoir potential” corresponds to the product of each species’ “specific infectivity” and larvae burden and host density. The “specific infectivity” is the proportion of nymphs that are infected produced by each infected species, i.e., a measure of species-specific transmission coefficient. As we did not have this type of data for the rodent species studied, we replaced the “specific infectivity” with the product of the “infectivity” and the infection prevalence of the population. The “infectivity” is defined as the proportion of all the nymphs that are infected that arise from an infected host. This infectivity generally is estimated in the laboratory [50]. We used data of infectivity found in the literature: 0.65 for bank voles and 0.54 for wood mice [51]. For Siberian chipmunks, we used the infectivity measured for the Eastern chipmunk (*Tamias striatus*), the most closely species for which there were data [52]: 0.44. As the time of repletion of feeding larvae on rodents is on average 3.5 days [53], we considered that the larvae burden on a host corresponded to a snapshot of the larvae burden of 3.5 consecutive days. In order to express the mean larvae burden of a host population by a representative unit of time in the formula of contribution, i.e., one day, we divided the estimated mean larvae burden by 3.5. For a given host population, we thus calculated the contribution to Lyme borreliosis risk as the number of infected questing nymphs produced per hectare per day with the formula (1):
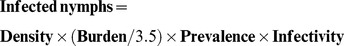
(1)with Infected nymphs, the number of infected questing nymphs produced per hectare and per day by the host species; Density, the estimated host density (number of individuals per hectare); Burden/3.5, the estimated mean larvae burden of the species per day; Prevalence, the estimated infection prevalence rate in *B. burgdorferi* sl or in *Borrelia* genospecies of the host (infection rate); and Infectivity, the species infectivity (rate). We simulated the distribution of each estimated contribution index to calculate 95% confidence intervals. To achieve this, each parameter (larvae burden, host density, infection prevalence) of the contribution index with the exception of infectivity, which is constant, were simulated independently according to their respective distribution centered on estimated mean values (Appendix S2). We tested if the contribution was different between rodent species (in 2007 and in 2008) and, from 2007 to 2010, for chipmunks, between seasons with sign tests (SIGN.test, library BSDA).

## Results

### Chipmunks *versus* Native Rodents in 2007 and 2008

For technical reasons, the tick load data and the prevalence data could not be obtained on exactly the same individuals. This is why the numbers of individuals with which larvae burden and infection prevalence are estimated are different (Table S1). Our approach was thus a population based approach and not an individual approach. In 2007, the mean density of chipmunks was respectively 2 and 1.5 times lower than the densities of voles and mice (Table 1). In 2008, the mean density of chipmunks was about 4 times lower than the density of voles (Table 1). For all of the rodents, the estimated larvae burdens were the lowest in the spring and the highest in the summer, where larvae burden reached 53.8 larvae/ind. for chipmunks in 2010 (Table 1). Siberian chipmunks were infected by three species of *B. burgdorferi* sl: *B. afzelii*, *B. burgdorferi* ss and *B. garinii-B. bavariensis,* where bank voles and wood mice all infected by *B. afzelii* except one wood mice infected by *B. burgdorferi* ss in June 2007. The *B. burgdorferi* sl overall, or the *B. afzelii* infection prevalence rate of the chipmunk population was most of the time higher than the one of bank voles and wood mice (Table 1, Table S1).

**Table 1:** Seasonal variation in estimated hosts density, *Ixodes ricinus* larval abundance, prevalence (Prev) of infection and contribution (Cont.) to the acarological risk of *Borrelia burgdorferi* genospecies for the different rodent host species between 2007 and 2010 on the Sénart Forest (France).

Species	Year	Seasons	Host density	*I. ricinus* larvae	*B. burgdorferi* s.s.		*B. afzelii*		*B. garinii*	
					Prev.	Cont.	Prev.	Cont.	Prev.	Cont.
Siberian chipmunks										
	2007	Spring	2 [2]–[3]	0.5 [0.3–0.6]	9 [4]–[21]	<0.1	22 [12]–[39]	<0.1	0	–
		Summer	8 [7]–[9]	28.6 [22.1–37.0]	7 [3]–[15]	2.0 [0.9–4.4]	10 [6]–[18]	3.0 [1.6–5.9]	0	–
		Autumn	3 [3]–[4]	23.6 [16.7–33.5]	16 [7]–[37]	1.7 [0.7–4.5]	33 [17]–[57]	3.4 [1.5–7.1]	<0.1	0
	2008	Spring	2 [1]–[3]	0.2 [0.2–0.3]	8 [4]–[18]	<0.1	12 [6]–[22]	0	<0.1	0
		Summer	5 [4]–[6]	13.0 [9.8–17.3]	6 [3]–[14]	0.5 [0.2–1.2]	5 [3]–[10]	0.5 [0.2–0.9]	0	–
		Autumn	5 [4]–[7]	10.8 [7.5–15.6]	15 [6]–[34]	1.1 [0.4–2.8]	19 [10]–[35]	1.4 [0.6–3.0]	<0.1	0
	2009	Spring	<1	0.4 [0.3–0.6]	2 [1]–[7]	<0.1	37 [19–63]	0	<0.1	0
		Summer	2 [1]–[3]	26.0 [18.3–36.9]	1 [0–5]	0.1 [0.0–0.4]	18 [10]–[34]	1.1 [0.5–2.5]	0	–
		Autumn	2 [1]–[3]	21.5 [14.1–32.8]	4 [1]–[14]	0.2 [0.1–0.9]	52 [27]–[8]	2.8 [1.3–5.9]	<0.1	0
	2010	Spring	<1	0.9 [0.6–1.4]	6 [1]–[23]	<0.1	4 [1]–[11]	<0.1	0	–
		Summer	4 [3]–[5]	53.8 [35.5–81.5]	4 [1]–[17]	1.1 [0.2–5.1]	2 [1]–[5]	0.5 [0.2–1.2]	0	–
		Autumn	7 [5]–[10]	44.5 [27.6–71.8]	10 [2]–[40]	2.8 [0.6–10.3]	7 [2]–[17]	1.8 [0.6–5.0]	0	–
Bank voles										
	2007	Spring	5 [1]–[10]	1.1 [0.7–1.4]	0	–	24 [16]–[35]	0.2 [0.1–0.6]	0	–
		Summer	12 [6]–[18]	5.6 [3.9–8.1]	0	–	23 [16]–[33]	2.8 [1.5–4.8]	0	–
		Autumn	9 [3]–[15]	1.8 [1.1–3.2]	0	–	17 [10]–[28]	0.5 [0.1–2.0]	0	–
	2008	Spring	5 [3]–[11]	0.4 [0.2–0.7]	0	–	8 [3]–[19]	<0.1	0	–
		Summer	12 [10]–[21]	1.1 [0.7–1.4]	0	–	3 [1]–[9]	0.1[0.0–0.2]	0	–
		Autumn	34 [27]–[46]	0.7 [0.4–1.4]	0	–	7 [2]–[21]	0.3 [0.1–0.9]	0	–
Wood mice										
	2007	Spring	8 [3]–[13]	1.4 [0.4–3.5]	0	–	0	–	0	–
		Summer	12 [6]–[18]	15.4 [6.3–38.2]	<0.1	<0.1	12.5*	3.4 [3.4–62.0]	0	–
		Autumn	0	–	–	–	–	–	–	–

Note: *B. garinii* includes *B. bavariensis;* Host density (estimation): number of individuals per hectare; *I. ricinus* larvae (estimation): mean number of larvae per individual; Prev. (estimation): mean percentage of infected individuals; Cont. (estimation): contribution in number of infected nymphs per hectare per day; *: observed prevalence; in brackets: confidence intervals at 95%.

**Figure 1:**
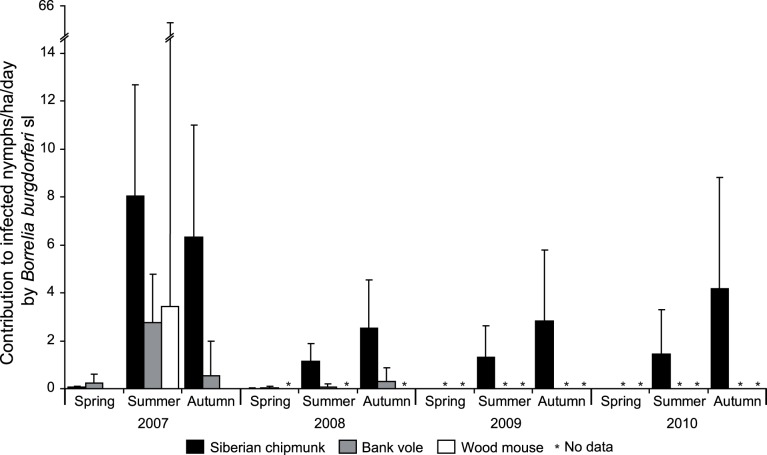
Estimated contributions to Lyme borreliosis (*Borrelia burgdorferi* sensu lato) risk of Siberian chipmunks, bank voles and wood mice. The contributions were estimated for Siberian chipmunks between 2007 and 2010, for bank voles in 2007 and 2008 and for wood mice in 2007, for 3 periods (spring, summer, autumn). Error bars are associated 95% confidence intervals.

Except in the spring, the Siberian chipmunk produced significantly more (about 8.5 times in 2007 and 2008) *B. burgdorferi* sl infected questing nymphs than the bank vole and the wood mouse (*P-values* <0.001). In 2007 and 2008, Siberian chipmunk population produced respectively 0.1 [95% confidence interval 0.0–0.1] and 0.0 [0.0–0.0] infected questing nymphs/ha/day in spring, 8.0 [4.9–12.7] and 1.1 [0.7–1.9] in summer, and 6.3 [3.5–11.0] and 2.5 [1.3–4.5] in autumn (Figure 1). The bank voles produced in 2007 and 2008 between 0.2 [0.1–0.6] and 2.8 [1.5–4.8] infected questing nymphs/ha/day. In 2007, the wood mice produced between 0.0 [0.0–4.7] and 3.4 [0.0–65.0] infected questing nymphs/ha/day (Figure 1). Similarly, except in the spring, Siberian chipmunks contributed also significantly more than bank voles to *B. afzelii* infected nymphs (*P-values* <0.001), but their contribution was similar to the one of wood mice in summer 2007 (*P-value = *0.73). Siberian chipmunks were the only contributors to *B. burgdorferi* ss infected nymphs (Table 1) and their contribution to *B. garinii-B. bavariensis* was closed to zero.

### Chipmunk Population from 2007 to 2010

From 2007 to 2010, chipmunk population densities varied between 0.2 and 7.8 ind./ha (Table 1). The mean larvae burden of the chipmunk population varied between 0.4 and 53.8 larvae/ind. from 2007 to 2010 (Table 1). The mean infection prevalence rate of the chipmunk population varied between 5 and 60% from 2007 and 2010 (Table 1). The contribution of Siberian chipmunks to Lyme borreliosis risk was overall 2.3 infected questing nymphs/ha/day. The index of contribution of the chipmunk population varied significantly between years (*P-values* <0.001, except between 2008 and 2009, for which *P-value* = 0.78), and between seasons (*P-values* <0.001). The contribution of chipmunks was 1.6 [1.1–2.3] infected questing nymphs/ha/day in 2007, 0.3 [0.2–0.5] in 2008, 0.3 [0.2–0.6] in 2009, and 0.5 [0.2–0.9] in 2010. Chipmunks produced a mean of 0.01 [0.01–0.03] infected questing nymphs/ha/day in spring, 2.3 [1.5–3.3] in summer, and 4.0 [2.4–6.5] in autumn. For *B. afzelii*, the contribution of chipmunks was 0.8 [0.5–1.3] infected questing nymphs/ha/day in 2007, 0.2 [0.1–0.3] in 2008, 0.3 [0.2–0.6] in 2009, and 0.2 [0.1–0.4] in 2010. Chipmunks produced a mean of 0.01 [0.003–0.01] *B. afzelii* infected questing nymphs/ha/day in spring, 1.0 [0.6–1.7] in summer, and 2.4 [1.2–4.4] in autumn.

## Discussion

Understanding how different host species contribute to the risk of vector-borne diseases is crucial in a context where the composition and structure of host communities are being modified worldwide [54]. Here, we provide a unique example of the quantification of the contribution to the risk of a zoonotic vector-borne disease, Lyme borreliosis, by an introduced species, the Siberian chipmunk. We showed that this introduced species contributes more to the risk than two native rodent reservoir hosts. Siberian chipmunks produced both *B. burgdorferi* ss nymphs and *B. afzelii* nymphs, whereas the other rodents produced only *B. afzelii* nymphs. We point out that the contribution varies significantly over time.

The contribution of the introduced Siberian chipmunks was higher – around 8.5 fold - than those of native reservoir rodents, mainly because of the chipmunk’s high tick burden and high infection prevalence compared to voles and mice. Chipmunks produced 80% of infected questing nymphs among the three rodent species, whereas voles and mice produced 10% each. The dominance (>65%) of one out of several rodent species in contributing to Lyme borreliosis risk also was found in the few other studies that quantified the contribution of different rodent species [5], [7], [55]. In these studies, the host species contributing most to the risk was either the most abundant species or that with the highest tick burden, infection prevalence, infectivity or a combination of these characteristics. The interesting finding in our study was that the dominant contribution of Siberian chipmunks was not due to its density, nor to its infectivity, both of which are lower than those of the native rodents. It was due instead to its high larvae burden and infection prevalence. Interestingly, O’Brien et al [13], the only other study of the amplification of a native pathogen by an introduced species, also showed that the amplification of Buggy Creek virus prevalence in native cliff swallows was due mainly to the higher infection prevalence of introduced house sparrows. The dominance of a host species in the contribution to Lyme borreliosis risk changed according to the composition of the community, but also according to the capacity of the different host species in a community to feed and infect ticks (our study). Furthermore, the contribution of a rodent species is lower when considering the whole host community rather than the rodents only. For instance, Brisson et al. [6] found that the white-footed mice contributed only 25% of the infected ticks produced and that the dominant species was the shrews (*Sorex cinereus* and *Blarina brevicauda*). Similarly, using vaccination, Tsao et al [56] showed that mice might contribute only to 55% of the infected larvae. Finally, the Siberian chipmunks have a larger size and a greater longevity compared to the native rodents. The larger size involves a larger surface of skin, which could increase their capacity to host ticks and thus their production of infected questing ticks in comparison to voles and mice. The greater longevity means that each individual chipmunk could contribute more to the acarological risk than voles and mice. This would be the case if chipmunks are able to maintain *Borrelia* during hibernation, which needs to be verified.

Not only do Siberian chipmunks contribute more than native rodents to the risk, they do so differently. The higher contribution of Siberian chipmunks was magnified by their infection by *B. burgdorferi* ss, that was not present in the native rodents, in addition to *B. afzelii*. The reservoir hosts for *B. burgdorferi* ss are not well known in Europe, but Sciurids are suspected to have an important role [22]. Unfortunately, in this study we were not able to differentiate between *B. garinii* and *B. bavariensis* that has been recently isolated in rodents [36]. Therefore our positive *B. garinii* could be either *B. bavariensis* or a new genotype of *B. garinii* associated with rodents.

The higher contribution of the introduced Siberian chipmunks to the risk compared to native rodents could be due to the additional infected nymphs produced by chipmunks; or to the production by chipmunks of nymphs that would otherwise have been produced by the native rodents, i.e., the chipmunks “feed” larvae, which originally were “intended” for the other rodents. The first hypothesis is the most plausible for several reasons. First, Siberian chipmunks produced additional nymphs infected by *B. burgdorferi* ss. Second, the larvae burden and infection prevalence of bank voles and wood mice in our study site were in accordance with observations made elsewhere in Europe where Siberian chipmunks were not present, with infection prevalence ranging between 9 and 28% [51], [57]–[59], and abundance ranging between 0.5 and 5 larvae/ind. for voles [60] and between 5 and 21 larvae/ind. for mice [61]. The additional production of infected nymphs by chipmunks would lead to the amplification of the circulation in native rodents of *B. afzelii*, the bacteria shared by all of the studied host species, through a phenomenon known as “spillback” [12]. It would increase the absolute risk for humans, since all of the *Borrelia* species involved are pathogenic for humans. To study this hypothesis, the contribution of voles and mice as well as the density of infected nymphs could be quantified on sites with and without chipmunks by taking into account potential confounding factors such as those influencing densities of ticks (e.g. roe deer (*Capreolus capreolus*) density, the most important host of adult ticks in Sénart).

The higher contribution of Siberian chipmunks is likely to persist over time, even though the contribution of chipmunks varied between years and seasons. Indeed both tick burden and infection prevalence of chipmunks have been shown to remain higher than those of bank voles over several years [23], [24], which is in accordance with our results in 2008. In addition, the population densities of bank voles and wood mice are subject to much harder crashes than those of Siberian chipmunks. For instance, we were not able to sample any native rodents in 2009, while chipmunks, although they also suffered a population crash [18], remained present on the study site. The seasonal variation of the Siberian chipmunk contribution followed that of larvae burden on hosts [5], [24]. The decrease of the contribution of chipmunks after 2008 depended mainly on the decrease in chipmunk densities. The low density in 2009 was explained by an important mortality of chipmunks during the winter of 2008–2009 that was connected with an absence of acorn production by oaks (*Quercus* spp.) during the previous autumn season (Chapuis J-L, unpublished data).

The parameters that were used to estimate the contribution (host density, larvae burden, infection prevalence and infectivity) vary according to time, space and the different populations. As far as we are aware, our study is one of the first to take into account the variability of the different parameters (with the exception of infectivity) in the estimation of the contribution. Studies based on similar estimations as ours [5], [7], [51], or those based on genetic assignation of *B. burgdorferi* sl [6], did not considered the variability of their parameters. Another source of variation could be linked to infectivity, which is susceptible to vary individually according to immune status. However, studying the infectivity variation in individuals requires very intensive work that must be conducted in a laboratory. Another option would be to use mixed models on repeated measures data to take into account this variability [38], or to use the “specific infectivity” proposed in Mather et al [7]. This, however, also requires heavy laboratory work as all individuals have to be maintained a few days in the laboratory. Furthermore, the infectivity for the Siberian chipmunk was based on data for the Eastern chipmunk [52]. To improve the calculation of our indexes, it would be necessary to characterize in the laboratory the infectivity of the Siberian chipmunk for the *B. burgdorferi* sl genospecies that it hosts.

We showed that the introduction of a host species to a local community can have important consequences on the risk for Lyme borreliosis. This means that it is not the level of biodiversity, but rather the species involved in a community, that most influences the risk of a multi-host disease [54]. Furthermore, our study adds a quantitative example to the effect of an introduced species on the dynamics of a local pathogen. To further investigate different species’ relative contribution to the risk of Lyme borreliosis, one should investigate the genetic variability of *B. burgdorferi* sl to find out how tight the strains are to the different hosts species. This should be done on housekeeping genes as well as on selected genes. This information could then be included in a dynamic model allowing the simultaneous integration of the vector population dynamics, the host population dynamics, and the dynamics of *B. burgdorferi* sl genospecies circulating in hosts [40]. Finally, an active survey around sites where Siberian chipmunks recently have been introduced could allow an investigation into whether the introduction is linked with an increase in the number of human cases.

## Supporting Information

Table S1 Observed larvae burden and infection prevalence in *B. burgdorferi* sensu lato in rodents collected from 2007 to 2010 on the Sénart Forest, France.
(DOCX)

Appendix S1 Correction of field tick counts for Siberian chipmunks to have laboratory tick counts.
(DOCX)

Appendix S2 Estimation of Confidence Intervals at 95% for the index of contribution.
(DOCX)
