# Supplementary material for: Introduced Siberian Chipmunks (Tamias sibiricus barberi) Contribute More to Lyme Borreliosis Risk than Native Reservoir Rodents
Source: PLoS One. 2013 Jan 31;8(1):e55377. doi: 10.1371/journal.pone.0055377 (PMC3561227; doi:10.1371/journal.pone.0055377)
Supplement: Table S1 — Observed larvae burden and infection prevalence in B. burgdorferi sensu lato in rodents collected from 2007 to 2010 on the Sénart Forest, France. (DOCX) [file pone.0055377.s001.docx]

**Supporting Information - Marsot et al. PLoS ONE 2013.**

**Table S1.** Observed larvae burden and infection prevalence in *B. burgdorferi* sensu lato in rodents collected from 2007 to 2010 on the Sénart Forest, France. Nb hosts: number of hosts examined; Nb larvae: mean number of *I. ricinus* larvae per host.

| **Species** | **Year** | **Season** | ***I. ricinus* larvae** | |  | **Prevalence (%)** | | | | |
| --- | --- | --- | --- | --- | --- | --- | --- | --- | --- | --- |
|  |  |  | nb hosts | nb larvae |  | nb hosts | *B. burgdorferi* s.s. | *B. afzelii* | *B. garinii* | *Overall B. burgdorferi* sl |
| Siberian chipmunk | | |  |  |  |  |  |  |  |  |
|  | 2007 | Spring | 82 | 1.4 |  | 17 | 29 | 41 | 0 | 71 |
|  |  | Summer | 211 | 52.6 |  | 62 | 13 | 19 | 0 | 45 |
|  |  | Autumn | 127 | 17.9 |  | 31 | 23 | 26 | 3 | 55 |
|  | 2008 | Spring | 16 | 0.4 |  | 57 | 18 | 12 | 2 | 32 |
|  |  | Summer | 126 | 18.1 |  | 103 | 8 | 9 | 3 | 19 |
|  |  | Autumn | 35 | 10.1 |  | 40 | 23 | 38 | 3 | 53 |
|  | 2009 | Spring | 0 |  |  | 3 | 0 | 67 | 0 | 67 |
|  |  | Summer | 9 | 75.7 |  | 16 | 6 | 19 | 13 | 31 |
|  |  | Autumn | 43 | 8.7 |  | 45 | 2 | 27 | 0 | 29 |
|  | 2010 | Spring | 7 | 0.6 |  | 6 | 17 | 17 | 0 | 33 |
|  |  | Summer | 90 | 65.8 |  | 90 | 4 | 4 | 0 | 9 |
|  |  | Autumn | 54 | 28.4 |  | 54 | 4 | 6 | 0 | 9 |
| Bank vole | | |  |  |  |  |  |  |  |  |
|  | 2007 | Spring | 68 | 1.0 |  | 83 | 0 | 24 | 0 | 24 |
|  |  | Summer | 108 | 5.5 |  | 107 | 0 | 23 | 0 | 23 |
|  |  | Autumn | 129 | 1.9 |  | 129 | 0 | 17 | 0 | 17 |
|  | 2008 | Spring | 65 | 0.4 |  | 73 | 0 | 8 | 0 | 8 |
|  |  | Summer | 86 | 0.9 |  | 91 | 0 | 3 | 0 | 3 |
|  |  | Autumn | 91 | 0.7 |  | 91 | 0 | 7 | 0 | 7 |
| Wood mouse | | |  |  |  |  |  |  |  |  |
|  | 2007 | Spring | 9 | 1.3 |  | 16 | 0 | 0 | 0 | 0 |
|  |  | Summer | 12 | 15.5 |  | 40 | <0.1 | 12 | 0 | 13 |
|  |  | Autumn | 13 | 7.8 |  | 16 | 0 | 6 | 0 | 6 |

Note: *B. garinii* includes *B. bavariensis*; nb hosts: sample size; coinfections are counted in both genospecies.
