# Supplementary material for: Introduced Siberian Chipmunks (Tamias sibiricus barberi) Contribute More to Lyme Borreliosis Risk than Native Reservoir Rodents
Source: PLoS One. 2013 Jan 31;8(1):e55377. doi: 10.1371/journal.pone.0055377 (PMC3561227; doi:10.1371/journal.pone.0055377)
Supplement: Appendix S1 — Correction of field tick counts for Siberian chipmunks to have laboratory tick counts. (DOCX) [file pone.0055377.s002.docx]

**Supporting Information - Marsot et al. PLoS ONE 2013.**

**Appendix S1:** Correction of field tick counts for Siberian chipmunks to have laboratory tick counts.

For the bank vole and the wood mouse, the data of tick burden were counts made in the laboratory on the whole body (almost exhaustive), and for the Siberian chipmunk, counts were made in the field only on the head. We thus estimated the number of ticks on chipmunks, which a laboratory count would have done, based on a count made in the field. One set of data realized in 2007 in Verneuil-sur-Seine (Yvelines) concerned 19 chipmunks, for which counts of ticks were realized at the same time in the field and in the laboratory. Counts were realized by a single experienced person and with high precision. The model corresponds to a linear model between field and laboratory data. The estimation thus was made from the field data, the only data available for chipmunks in our study. The explained variable is thus Y_F_ (number of larvae counted in the field) and the explanatory variable Y_L_ (number of larvae *I. ricinus* found by an individual in the laboratory).

The count variables for every individual i, are respectively:

Y_L_^i^~ Poisson (λ_L_^i^) with E(Y_L_^i^)=Var(Y_L_^i^)= λ_L_^i^ , and

Y_F_^i^~ Poisson (λ_T_^i^) with E(Y_T_^i^)=Var(Y_T_^i^)= λ_T_^i^ .

By taking into account the body surface of every animal i, on which ticks are counted, we obtained:

λ_L_^i^= μ_L._S^i^ (S^i^ entire body surface of the animal i, μ_L_ mean density of ticks) [1]

λ_F_^i^= μ_F ._s^i^ (s^i^ head surface of the animal i, μ_F_ mean density of ticks) [2]

We hypothesized that there is a linear relation between μ_L_ et μ_F_:

μ_F_ = a.μ_L_ [3]

where a took into account the difference of density evaluated in the field and in the laboratory and the aggregation of ticks on the head. By using equations [1], [2] and [3], we obtained:

λ_F_^i^ = a.R_i_ .λ_L_^i^ with R_i =_ (s^i^/ S^i^)

We hypothesized that, for a given host species, the rate R_i_ is constant and did not depend on the animal i: R_i_ = R

Thus, we obtained:

λ_F_^i^ = a.R.λ_L_^i^ [4]

In the absence of independent measures of a and R, it is not possible to estimate separately these parameters. Thus, we said: α = a.R. With the equation [4], the mean counts in the field and in the laboratory were linked by the expression:

**λ_F_^i^ = α.λ_L_^i^** [5]

Z_L_^i^ = √Y_L_^i^ and Z_F_^i^ = √ Y_F_^i^ represent the square root of the initial counts. As Y_L_ and Y_F_ followed a Poisson distributions, Z_L_ and Z_F_ followed normal distributions [[27](#_ENREF_27)], such as:

Z_L_^i^ ~ Normal (√λ_L_^i^, ¼) and

Z_F_^i^ ~ Normal (√λ_F_^i^, ¼).

After standardization of Z_L_ et Z_F_, we obtained:

Z_L_^i^ = √ λ_L_^i^ + ε_1_ with ε_1_ ~ Normal (0,1/4) and

Z_F_^i^ = √ λ_F_^i^ + ε_2_ with ε_2_ ~ Normal (0,1/4).

With the equation [5], we obtained:

√ **λ_F_^i^ =** √**α .**√ **λ_L_^i^**

which allowed us to obtain a model of estimation:

Z_F_^i^ = √ α .Z_L_^i^ + [ε_2_ – (ε_1_.√ α)] with ε_1_ et ε_2_ independents.

With e = ε_2_ – (ε_1_.√ α), we had E(e) = 0 and Var(e) = ¼ .(α+1), thus an estimation model:

**Z_F_^i^ = √ α .Z_L_^i^ + e^i^**

It corresponded to a classic linear model of regression, which did not require the estimation of an intercept. We obtained $\hat{\alpha}$=² and $\hat{\sigma}$²_residual_= ¼. [$\hat{\alpha}$ + 1], where represented the estimation of the slope and $\hat{\sigma}$²_residual_ the residual variance of the regression model.
