# Supplementary material for: Introduced Siberian Chipmunks (Tamias sibiricus barberi) Contribute More to Lyme Borreliosis Risk than Native Reservoir Rodents
Source: PLoS One. 2013 Jan 31;8(1):e55377. doi: 10.1371/journal.pone.0055377 (PMC3561227; doi:10.1371/journal.pone.0055377)
Supplement: Appendix S2 — Estimation of Confidence Intervals at 95% for the index of contribution. (DOCX) [file pone.0055377.s003.docx]

**Supporting Information - Marsot et al. PLoS ONE 2013.**

**Appendix S2**: Estimation of Confidence Intervals at 95% for the index of contribution

### # Simulation function for estimation of confidence intervals of indexes of contribution

FctSim <- function(vch, vde, vpr, pin) {

# vx : (mean, s.e.)

Nsim <- 1000

ySim <- rep(0.0,Nsim)

for ( i in 1:Nsim ) {

# larvae burden

z <- rnorm(1,vch[1],vch[2])

y1 <- exp(z)

# host density

z <- rnorm(1,log(vde[1]),

sqrt(((1.0/vde[1])**2)*(vde[2]**2)) )

y2 <- exp(z)

# infection prevalence

z <- rnorm(1,vpr[1],vpr[2])

y3 <- 1 - (exp(-exp(z)))

# calcul indice

ySim[i] <- y1*y2*y3*pin

}

# Example for Siberian chipmunk in spring 2007

set.seed(1000)

a1 <- 0.8

a2 <- 0.2

b1 <- 2.4

b2 <- 0.4

c1 <- 0.58

c2 <- 0.24

d <- 0.44

z <- FctSim(c(a1,a2),c(b1,b2),c(c1,c2),d)

index <- (exp(a1)*b1*(1-(exp(-exp(c1))))*d)/3.5

index

# 0.05812287

IC <- quantile(z,probs=c(0.025,0.975))

IC

# 2.5% 97.5%

# 0.0310401 0.1072457
